# Identification and analysis of the complete mitochondrial genome of *Phyllotreta striolata* (Coleoptera, Chrysomelidae)

**DOI:** 10.1080/23802359.2019.1622469

**Published:** 2019-07-10

**Authors:** Zhenhua Zu, Chengjin Yan

**Affiliations:** Institute of Agriculture and Biotechnology, Wenzhou Vocational College of Science and Technology, Wenzhou, China

**Keywords:** Chrysomeloidea, complete mitochondrial genome, *Phyllotreta striolata*

## Abstract

In this study, the complete mitochondrial genome of *Phyllotreta striolata* (Coleoptera, Chrysomeloidea, Chrysomelidae) was first determined. The complete genome is 15,689 bp in length. It contains 13 protein-coding genes (PCGs), 22 tRNA genes, two rRNA genes, and a control region (A + T-rich region). The gene organization, nucleotide composition, and codon usage are similar to other Chrysomelidae mitogenomes. The overall nucleotide composition was 39.90% A, 35.94% T, 15.27% C, and 8.89% G, respectively. Phylogenetic analysis both highly supported that *P. striolata* showed a close relationship with *P. undulata*. The measure of complete mitogenome sequence of *P. striolata* will provide fundamental data for the phylogenetic and biogeographic studies of the Chrysomeloidea and Coleoptera.

The striped flea beetle (SFB), *Phyllotreta striolata* (Fabricius) (Coleoptera, Chrysomelidae, Galerucinae), is a notorious vegetable pest worldwide. It is harmful to many plants, especially cruciferous vegetables (Feeny et al. 1970). In the south of China, the cultivation area of cruciferous vegetables is expanding year by year, which results in the continuous occurrence of the striped flea beetles. In addition, the long-term use of a single chemical control method in production has led to rapid and widespread improvement of pesticide resistance in the population of *P. striolata*, and its occurrence is becoming more and more serious (Liu et al. 2018). So, it is important to sequence and annotate the mitochondrial genome (mito-genome) of *P. striolata*. Here, we report the complete mito-genome sequence of *P. striolata* by next-generation sequencing (NGS). The annotated mitochondrial DNA (mtDNA) sequence has been deposited in GenBank under accession number MK256930.

In this study, the specimens were collected from Wenzhou, Zhejiang province (28°5′18.11″N, 120°30′55.57″E) of China in September 2018 and were deposited in the insect collection of Wenzhou Vocational College of Science and Technology (Wenzhou, China).

The specimens were initially preserved in 100% ethanol and then stored at 4 °C prior to DNA extraction. Total genomic DNA was extracted from the individual adult specimen using a DNeasy tissue kit (Qiagen, Hilden, Germany) following the manufacturer’s protocol. One library was constructed with VAHTS™ Universal DNA Library Prep Kit for Illumina^®^ and sequenced with Illumina HiSeq X Ten sequencer (150 bp pared-end) by Novogene (Beijing, China).

The complete mitochondrial genome of *P. striolata* was a circular molecule with 15,689 bp in length (GenBank Accession number MK256930). The mitogenome of *P. striolata* contained two rRNA genes, 13 protein-coding genes (PCGs), 22 tRNA genes, and a control region. The overall nucleotide composition was 39.90% A, 35.94% T, 15.27% C, and 8.89% G, with a slight AT bias of 75.84%. Five PCGs initiation codons were ATT, four PCGs initiation codons were ATG, two PCGs initiation codons were ATA, two PCGs began with ATC, different from *P. undulata* (*cox2*, *nad4l*, and *nad6* with ATA, *nad4* with ATG, *cob* with ATT) and *P. parallela* (*cox2* and *nad5* with ATA, *nad4* with ATG, *cob* with ATC) (Gomez-Rodriguez et al. 2015). Correspondingly, six PCGs stopped with the complete termination codon TAA, two PCGs stopped with the complete termination codon TAG, *cox2* stopped with an incomplete termination codon TA while the rest of PCGs ended with an incomplete termination codon T–– (*atp8*, *cox 3*, *nad4*, and *nad5*), which was different from *P. undulata* and *P. parallela* (*nad2, nad3*, and *cox1* with T, *atp8* and *cox3* with TAA) (Gomez-Rodriguez et al. 2015). Moreover, the 22 tRNA genes ranged in size from 59 bp (tRNA^Ser(UCU)^) to 73 bp (tRNA^Lys^). The two rRNA genes of *P. striolata* were determined by sequence alignment with other species. The *lrRNA* is located between the tRNA^Leu(UAG)^ and tRNA^Val^ with a 1262bp in length while the *srRNA* is located between the tRNA^Val^ and the control region with a 746 bp in length.

To confirm the phylogenetic relationships between *P. striolata* and other Chrysomelidae, phylogenetic analysis was performed on the concatenated dataset of 13 PCGs at neighbour-joining (NJ) method that produced NJ tree (Gomez-Rodriguez et al., 2015; Nie et al., 2018; Zhang et al., 2017) (Figure 1). *Sitona callosus* (Curculionidae, Entiminae) was defined as an outgroup. The other nine species were divided into two clades. *Phyllotreta parallela*, *P. undulata* and *P. striolata* were grouped in one clade, suggested the close relationship of these species, and further confirmed that *P. striolata* belongs to the subfamily Galerucinae. *Phyllotreta striolata* was evolutionarily closest to *P. undulata*, and *P. parallela* was a sister of *P. striolata* (Figure 1).

**Figure 1.**
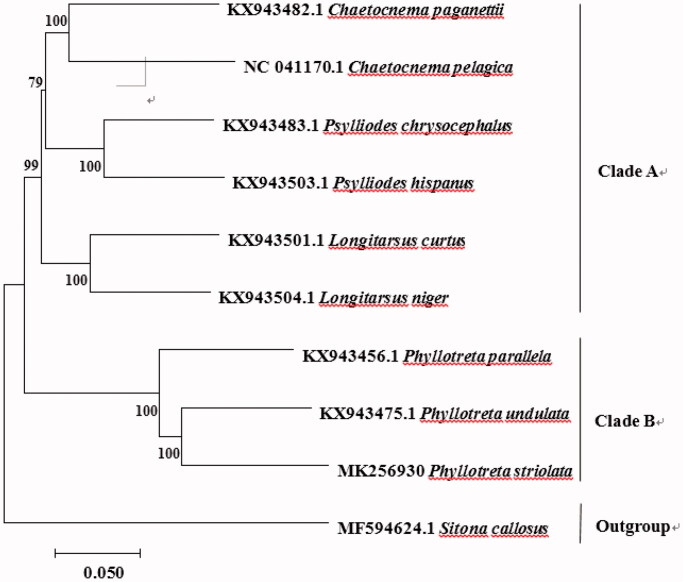
Neighbour-joining (NJ) phylogenetic tree of *Phyllotreta striolata* and nine other species using *Sitona callosus* as an outgroup. The number above each node indicates the NJ bootstrap support values.
